# *Anopheles gambiae s.s.* resistance to pyrethroids and DDT in semi-urban and rural areas of the Moyen-Ogooué Province, Gabon

**DOI:** 10.1186/s12936-023-04820-y

**Published:** 2023-12-18

**Authors:** Stravensky Térence Boussougou-Sambe, Barclaye Ngossanga, Ange Gatien Doumba-Ndalembouly, Lynda Nouage Boussougou, Tamirat Gebru Woldearegai, Fabrice Mougeni, Thierry Ndong Mba, Jean Ronald Edoa, Jean Claude Dejon-Agobé, Parfait Awono-Ambene, Peter G. Kremsner, Hilaire M. Kenguele, Steffen Borrmann, Benjamin Mordmüller, Ayôla Akim Adegnika

**Affiliations:** 1 Centre de Recherches Médicales de Lambaréné, Lambaréné, Gabon; 2 Institut für Tropenmedizin, Eberhard-Karls-Universität, Tübingen, Germany; 3 Université des sciences et techniques de Masuku, Masuku, Gabon; 4 Institut de Recherche de Yaoundé, Organisation de Coordination pour la lutte contre les Endémies en Afrique Centrale (OCEAC), B.P. 288 Yaoundé, Cameroon; 5 German Center for Infection Research (DZIF), Partner Site, Tübingen, Germany; 6 Department of Medical Microbiology, Radboudumc, Nijmegen, The Netherlands; 7 Fondation pour la Recherche Scientifique (FORS), Cotonou, Benin

**Keywords:** *Anopheles gambiae*, Pyrethroids, Organochlorides, Insecticide resistance, Moyen Ogooué Province, Gabon

## Abstract

**Background:**

Pyrethroids are the main insecticides used in vector control for malaria. However, their extensive use in the impregnation of long-lasting insecticidal nets (LLINs) and indoor residual spraying has led to the development of resistance, threatening its success as a tool for malaria control. Baseline data prior to large scale distribution of LLINs are important for the implementation of efficient strategies. However, no data on the susceptibility of malaria vectors is available in the Moyen-Ogooué Province in Gabon. The aim of this study was to assess the susceptibility to pyrethroids and organochlorides of malaria vectors from a semi-urban and rural areas of the province and to determine the frequency of insecticide resistance genes.

**Methods:**

Larvae were collected from breeding sites in Lambaréné and Zilé and reared to adults. Three to five-day old female *Anopheles gambiae sensu lato* mosquitoes were used in cone tube assays following the WHO susceptibility tests protocol for adult mosquitoes. A subsample was molecularly identified using the *SINE200* protocol and the frequency of *Vgsc-1014 F* and − *1014 S* mutations were determined.

**Results:**

*Anopheles gambiae sensu stricto* (*s.s*.) was the sole species present in both Lambaréné and Zilé. Mosquito populations from the two areas were resistant to pyrethroids and organochlorides. Resistance was more pronounced for permethrin and DDT with mortality lower than 7% for both insecticides in the two study areas. Mosquitoes were statistically more resistant (*P <* 0.0001) to deltamethrin in Lambaréné (51%) compared to Zilé (76%). All the mosquitoes tested were heterozygous or homozygous for the knockdown resistance (*Kdr*) mutations *Vgsc-L1014F* and *Vgsc-L1014S* with a higher proportion of *Vgsc-L1014F* homozygous in Lambaréné (76.7%) compared to Zilé (57.1%).

**Conclusion:**

This study provides evidence of widespread resistance to pyrethroids in *An. gambiae s.s.*, the main malaria vector in the Moyen-Ogooué Province. Further investigation of the mechanisms underlining the resistance of *An. gambiae s.s.* to pyrethroids is needed to implement appropriate insecticide resistance management strategies.

## Background

Vector control has been pivotal for malaria control, with long-lasting insecticidal nets (LLINs) and indoor residual spraying (IRS) being the two main strategies used. It is estimated that these intervention especially LLINs have averted 69% of the 663 million malaria cases [1]. Five classes of insecticides are used in vector control: (1) pyrethroids, (2) carbamates, (3) organophosphates, (4) organochlorides and (5) chlorfenapyr, a pyrrole which recently received an interim approval from the World Health Organization (WHO) to be used in insecticide-treated nets and in IRS [2].

However, up to now, insecticides of the pyrethroid class are mostly used for impregnating bed nets. This reliance on a sole class of insecticides has led to the development and spread of resistance in major *Anopheles* vectors in Africa [3–7], especially following mass distribution of LLINs [8]. Two mutations (*L1014F* and *L1014S*) at the domain II of the voltage-gated sodium channel gene (*Vgsc*) have been identified as providing cross-resistance to pyrethroids and DDT in *Anopheles gambiae sensu stricto* (*s.s*.) [9, 10]. Although the consequences of this resistance on vector control measures are not fully elucidated, some studies have shown a loss of efficacy of LLINs in areas with insecticide resistance [4, 11, 12].

Malaria remains a public health issue in Gabon where it is a primary reason for consultation. Malaria prevention in Gabon is based on the provision of intermittent preventive treatment with sulfadoxine-pyrimethamine (IPTp-SP) to pregnant women who are in addition provided LLINs which are also freely available to children under five [13]. Despite the absence of LLIN mass distribution, previous studies have reported high frequencies of the *Vgsc-L1014F* and *Vgsc-L1014S* mutations in *Anopheles* populations from Libreville, Port-Gentil, Mouila and in villages of the Moyen-Ogooué province (Bindo, Zilé and Nombakélé) and in a lower frequency in Benguia [14–18]. In fact, Libreville was the first coastal West African location where the presence of both *Vgsc-L1014F* and *Vgsc-L1014S* alleles was reported suggesting the presence of high level of resistance in this mosquito population [19]. This may need re-evaluation of the effectivity of LLINs for the control of malaria morbidity in the country. However, only a single study assessed the phenotypic resistance in mosquitoes populations in Gabon [17] and there is a lack of data on the susceptibility of malaria vectors to insecticides from many parts of the country.

The aim of this study was to assess the susceptibility to pyrethroids and organochlorides of malaria vectors from two different settings of the Province of Moyen-Ogooué and to determine the frequency of insecticide resistance genes.

## Methods

### Study areas

**Fig. 1:**
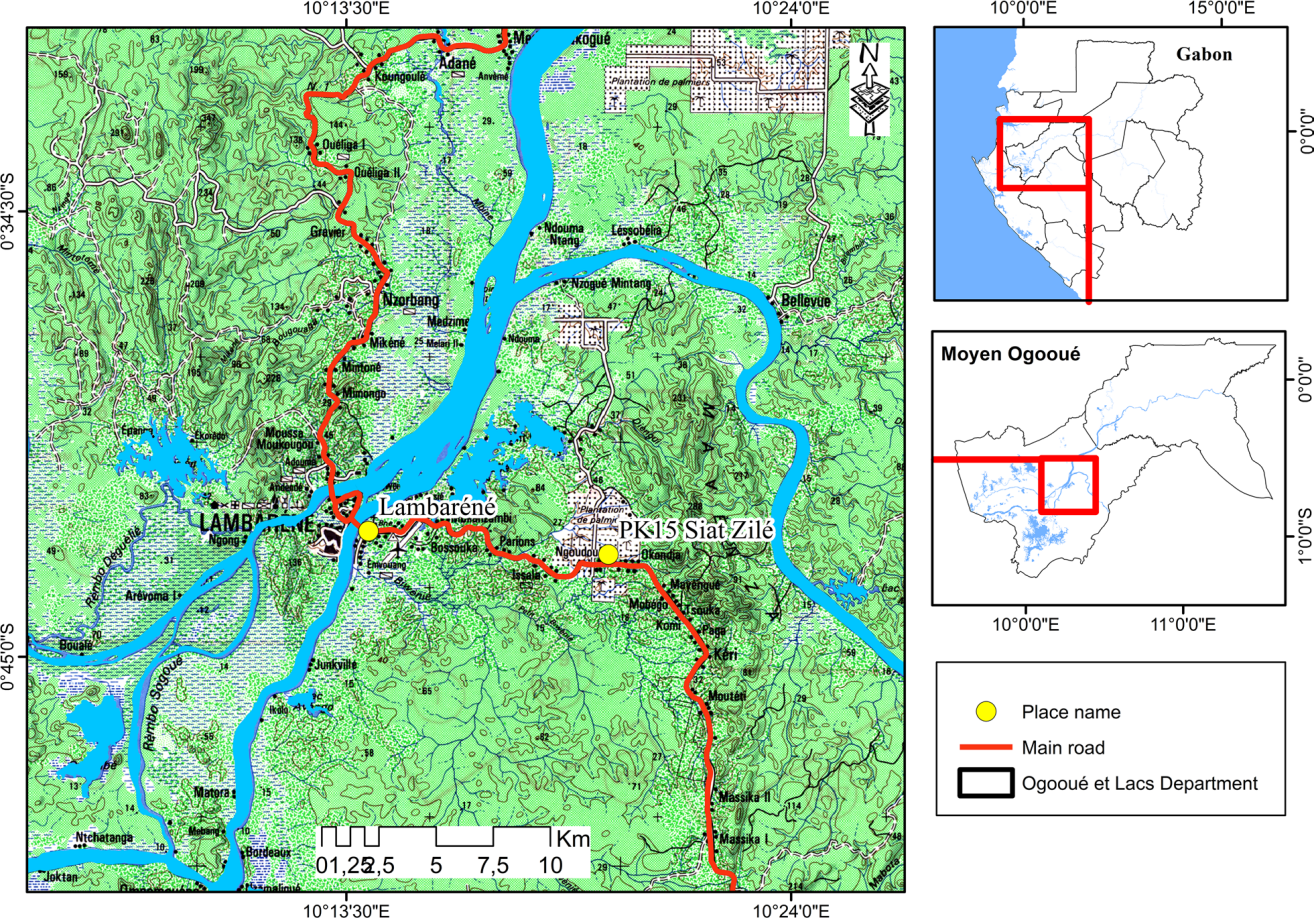
Map of the studied areas

The study was conducted in Lambaréné and Zilé from November 2017 to February 2018 (Fig. 1). Lambaréné is a semi urban area and is the provincial capital of the Moyen-Ogooué Province while Zilé is a rural area located approximately 12 km from Lambaréné. Although the two areas are close, a previous study in the Zilé area has showed that malaria transmission in this area is perennial with a fixation of the *Vgsc-L1014F* and *Vgsc-L1014S* resistant alleles in *An. gambiae s.s*. populations [18]. In addition, Zilé has the particularity of housing a rubber plantations scheme with the potential use of insecticides to deal with plant pests (although not assessed here) potentially driving the resistance of *An. gambiae sensu lato (s.l.)* populations.

### Mosquito collection

Open water bodies in the areas were explored for the presence of anophelinae larvae by dipping and larvae were collected from breeding sites with the presence of larvae in the two areas. The larvae were reared up to the adult stage at the Medical Entomology Laboratory of the Centre de Recherches Médicales de Lambaréné (CERMEL). Emerging mosquitoes were provided with 10% sugar solution until the day of the testing. Adult mosquitoes were identified using the morphological identification keys of Gillies and de Meillon [20] and Gillies and Coetzee [21].

### WHO susceptibility assays

The tests were carried out using impregnated papers with the following diagnostics concentrations: deltamethrin (0.05%), permethrin (0.75%) and DDT (4%) produced by the Vector Control Research Unit of Sains Malaysia University (Penang, Malaysia) and kindly provided by the Organisation de Coordination pour la lutte contre les Endémies en Afrique Centrale (OCEAC). The impregnated papers were tested with the Kisumu strain reared at the CERMEL before the tests were conducted with field collected mosquitoes to assess their quality. The results from the tests with the Kisumu were also used as comparators to the knockdown times obtained with field populations.

The tests were carried out according to the WHO protocol [22]. Briefly, three-to-five-day old, starved *An. gambiae s.l.* mosquitoes were exposed in WHO susceptibility kits to impregnated papers with insecticides while controls mosquitoes were exposed to untreated filter papers. The number of mosquitoes knocked down was recorded at 5, 10, 15, 20, 30, 40, 50 and 60 min. After 1 h of exposure, mosquitoes were transferred to observation tubes and were maintained on a 10% sugar solution for 24 h. Mortality was recorded after a 24-hr recovery period. The mosquitoes were stored on silica gel for molecular assays.

### Molecular identification

DNA was extracted from randomly selected control mosquitoes from Lambaréné and Zilé using the Livak protocol [23]. The mosquitoes were identified using the *SINE200* protocol [24]. This protocol allows for the simultaneous identification of members of the *An. gambiae* complex using the following set of primers: forward (5′-TCG CCT TAG ACC TTG CGT TA-3′) and reverse (5′-CGC TTC AAG AAT TCG AGA TAC-3′) primers. The cycling conditions were as follows: initial denaturation at 95 °C for 5 min, followed by 35 cycles at 95 °C for 30 s, annealing at 54 °C for 30 s, extension at 72 °C for 1 min and final extension at 72 °C for 10 min. The PCR products were analysed on a 2% agarose gel.

### *kdr* genotyping

A Taqman assay was used to determine the frequency of *kdr* mutations (*Vgsc-L1014F* and *L1014S*) in dead and alive mosquitoes based on the protocol from Bass et al. [25]. The protocol is based on the use of one set of primers (5′-CAT TTT TCT TGG CCA CTG TAG TGA T-3′; *kdr-*reverse: 5′-CGA TCT TGG TCC ATG TTA ATT TGC A-3) and three probes: one for the identification of the wild-type allele (5′-CTT ACG ACT AAA TTTC-3′) labelled with the HEX fluorophore, and the remaining two labelled with the FAM fluorophore for the identification of the *Vgsc-L1014F* mutation (5′-ACG ACA AAA TTT C-3′) and the *Vgsc-L1014S* mutation (5′-ACG ACT GAA TTT C-3′). The cycling conditions consisted of an initial denaturation at 95 °C for 10 min, followed by 40 cycles at 95 °C for 10 s and 65 °C for 45 s.

### Statistical analysis

The WIN DL (version 2.0, 1999) software was used to determine the different knockdown times for 50 and 95% tested samples knockdown times from each population (KDT_50_, KDT_95_) using a log-time probit model.

Mortality was calculated by dividing the proportion of dead mosquitoes after the 24 h recovery period to mosquitoes exposed to the insecticide. The results of the test were interpreted based on the WHO guidelines [26] with mortality in the range 98–100% indicating susceptibility, while 90–97% mortality indicating potential resistance and a need to be further investigated. Mortality rates of less than 90% were interpreted as resistance.

The sample size of mosquitoes to be analysed for the molecular identification was based on an estimated proportion of *An. gambiae s.s.* equals to 98%. The following formula: n = ε^2^ [p (1-p)]/e^2^; with ε = 1.96 (alpha risk = 5%), e (precision) = 5% and p = expected prevalence; with the resulting n = 31 to be included from each site.

The differences in mortality and allelic frequencies in the mosquito populations from the two areas were compared using the Fisher’s exact test with the R software v.3.2.5 [26]. In addition, the distribution of genotypes was also tested for conformity to Hardy Weinberg Equilibrium (HWE) within each site using a web-based tool (https://gene-calc.pl/hardy-weinberg-page).

## Results

A total of 537 *An. gambiae s.l.* collected from Lambaréné and Zilé were tested in susceptibility assays. In Lambaréné, 85 mosquitoes were tested with permethrin, 130 mosquitoes with deltamethrin and 44 with DDT. Meanwhile in Zilé, 113 mosquitoes were tested with permethrin, 117 mosquitoes with deltamethrin and 99 with DDT.

### Species composition

Out of the 116 *An. gambiae s.l.* that were identified molecularly, 54 out of 60 mosquitoes and all mosquitoes (56 mosquitoes) were successfully amplified in Lambaréné and Zilé, respectively. *Anopheles gambiae* *s.s.* was the sole species found in both Lambaréné and Zilé.

### Knockdown times

The KDT_50_ and KDT_95_ could only be determined for deltamethrin while for permethrin and DDT, both were above 60 min. The KDT_50_ for deltamethrin were around 32 min and 30 min for *An. gambiae* from Lambaréné and Zilé, respectively (Table 1). When compared to the KDT_50_ of the susceptible Kisumu strain, these KDT_50_ represented a 3.4 and 3.1-fold increase in the amount of time required to knockdown 50% of the *An. gambiae s.s.* mosquitoes from Lambaréné and Zilé, respectively. Similarly, there was an increase in KDT_95_ (58 min in mosquitoes from Lambaréné and 48 min in Zilé) for deltamethrin when compared to the one from the susceptible Kisumu strain, representing a 2.6 and a 1.6-fold increase.

**Table 1:** Knockdown times and ratios of *An. gambiae s.s.* samples from Lambaréné and Zilé after insecticide susceptibility tests

Study sites	Insecticides tested	N	KD_50_(min) [CI_95_]	Rtkd_50_ [CI_95_]	KD_95_ (min) [CI_95_]	Rtkd_95_ [CI_95_]	Status
Lambaréné	Del 0.05%	130	32.3 [30.8–33.7]	3.4	57.7 [54 -62.7]	2.6	Resistant
	Per 0.75%	85	> 60	NA	> 60	NA	Resistant
	DDT 4%	44	> 60	NA	> 60	NA	Resistant
Zilé	Del 0.05%	117	29.9 [28.6–31.1]	3.1	48.1 [45.4–51.7]	1.6	Resistant
	Per 0.75%	113	> 60	NA	> 60	NA	Resistant
	DDT 4%	99	> 60	NA	> 60	NA	Resistant

### Insecticide susceptibility of *An. gambiae s.s.*

**Fig. 2:**
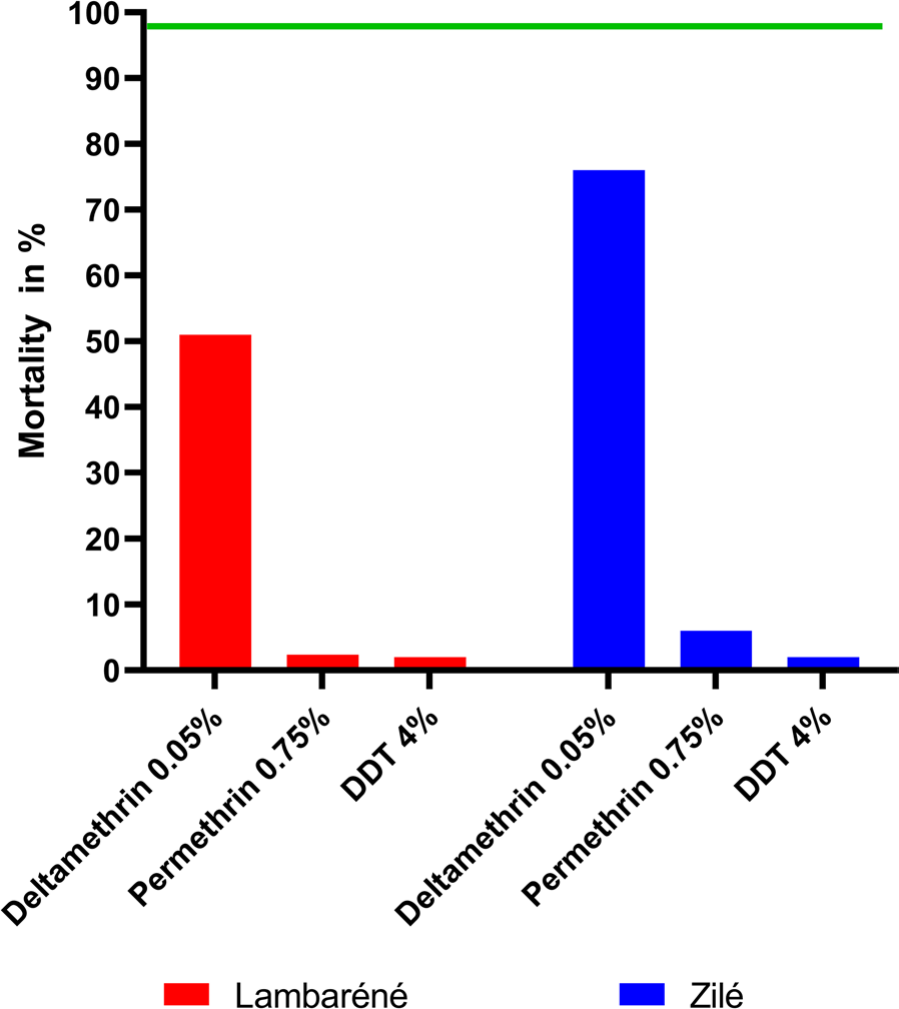
Mortality rates of *An. gambiae s.s.* from Lambaréné and Zilé exposed to deltamethrin, permethrin and DDT. The green line represents the threshold for full susceptibility according to the WHO criteria

The mosquitoes from Lambaréné and Zilé were found to be resistant to permethrin, deltamethrin, and DDT but the resistance was more pronounced for permethrin and DDT (Fig. 2). The mortalities recorded after the 24 h recovery period with permethrin and DDT were below 3% for both insecticides in Lambaréné. Whilst in Zilé, mortality with permethrin and DDT were 6% and 2%, respectively. The mortality rates for permethrin (Fisher’s exact test, *P* = 0.30) and DDT (Fisher’s exact test, *P* = 1) were comparable in both study areas. *Anopheles gambiae s.s.* mosquitoes from Lambaréné were significantly more resistant to deltamethrin (Fisher’s exact test, *P <* 0.0001) than those from Zilé, with mortalities of 51% and 76%, respectively.

### Genotypic resistance markers

Out of the 118 mosquitoes that were randomly screened for *kdr* mutations, 116 (98%) were successfully amplified. All the mosquitoes were heterozygous (80.2%) or homozygous (19.8%) for *Vgsc-L1014F* and *-L1014S* which confer resistance to pyrethroids and DDT. The distribution of the resistant genotypes were similar in Lambaréné and Zilé (Fisher’s exact test, *P* = 0.068) with a higher proportion of homozygous *Vgsc-L1014F* (76.7% and 57.1%, respectively), followed by heterozygous individuals *Vgsc-L1014F/L1014S* (20% and 32.1%, respectively) with the rest made of homozygous *Vgsc-L1014S* (Table 2) (2% and 6%, respectively). However, the distribution of the genotypes were consistent with HWE in Lambaréné (χ^2^ = 1.09; p = 0.58) and Zilé (χ^2^ = 1.82; p = 0.40). None of the mosquitoes were found to carry the susceptible allele *Vgsc-L1014L.*

**Table 2:** Frequencies of *knock-down resistance* (*kdr*) alleles *An. gambiae s.s.* from Lambaréné and Zilé

	N	Genotypic frequencies n (%)			Allelic frequencies n (%)		HWE
		*L1014F/L1014F*	*L1014F/ L1014S*	*L1014S/L1014S*	*L1014F*	*L1014S*	
Lambaréné	60	46 (76.7)	12 (20)	2 (3.3)	52 (86.7)	8 (13.3)	0.58
Zilé	56	32 (57.1)	18 (32.1)	6 (10.8)	41 (73.2)	15 (26.8)	0.40
Total	116	78 (67.2)	30 (25.9)	8 (6.9)	93 (80.2)	23 (19.8)	0.13

p-values for chi-square test of Hardy Weinberg equilibrium

## Discussion

Vector controls measures should be implemented based on the local epidemiological and entomological data. Thus, there is a need to determine the susceptibility of local vectors to the common types of insecticides before large vector control measures are deployed in an area. This report provides baseline data on the phenotypic susceptibility of malaria vectors in the Moyen-Ogooué Province. Results from the current study revealed a substantial increase of KDT_50_ and KDT_95_, when compared to the Kisumu susceptible strain, beyond 60 min for permethrin and DDT. Whereas for deltamethrin, although KDT_50_ and KDT_95_ were below 60 min, it represented a 3-fold increase in KDT_50_ and up to a 2.6- fold increase in KDT_95_ when compared to the susceptible *An. gambiae s.s.* Kisumu strain. Similar increases in KDT_50_ were also recorded in susceptibility tests carried out in Mouila, the capital of the Ngounié province of Gabon [17]. This loss of susceptibility to the knockdown effect of pyrethroids may lead to the loss of the deterrence effect to this class of insecticide which are used in impregnating bed nets. This deterrence effect is of great importance in maintaining LLINs effectiveness especially when they are torn [11] as they prevent entry of mosquitoes. These results suggest that pyrethroid based insecticides are widely used in the local populations.

The mortality rates recorded for each of the insecticides tested are in line with the knockdown results suggesting *An. gambiae s.s.* from the area are highly resistant to pyrethroids and DDT. *Anopheles gambiae s.s.* populations were highly resistant to DDT and permethrin, a type I pyrethroid. Previous reports from other countries [5, 7, 27, 28] have shown a high resistance to permethrin, which is, together with deltamethrin, the main insecticides used in bed nets impregnation. Resistance to deltamethrin, a type II pyrethroid, was less pronounced than for permethrin thus LLINs impregnated with deltamethrin may be a better option for vector control in the Moyen-Ogooué Province and by extension to Gabon, as the current results are in line with those found in the Ngounié province for permethrin and deltamethrin [17].

The resistance in this study was more pronounced in the urban compared to the rural area despite the fact that the larval collection in the latter was carried out in an area surrounded with rubber and palm oil plantations where insecticides, such as pyrethroids, may be used for pest control as reported in agricultural settings in Burkina Faso [29, 30]. However, from discussions held with personnels from both rubber and palm oil plantations, insecticides are not used for pest controls with the plants only sprayed with the herbicide glyphosate which is not linked to pyrethroids resistance. Therefore, the personal use of pyrethroids based vector control tools in the form of insecticide sprays, mosquito coil, impregnated nets could play a major role in the selection pressure of local *Anopheles* spp for reduced susceptibility to pyrethroids especially in urban areas. This high level of resistance could lead to the failure of control measures to reduce malaria transmission especially using bed nets that are impregnated with pyrethroids. These results point to the need for the implementation of mitigation strategies such as the use of LLINs impregnated with compounds such as piperonyl butoxide (PBO), which inhibits cytochrome P450, involved in metabolic resistance, and thereby can restore pyrethroid susceptibility.

Increases in KDT_50_ in field mosquito populations has been suggested to provide a sensitive indicator of the implication of *kdr* mutations in phenotypic resistance to pyrethroids [22, 31]. The *kdr* mutations in the local populations are fixed with all *An. gambiae s.s.* carrying either the *Vgsc-L1014F* or *Vgsc-L1014S* mutations which may explain the loss of knockdown effects of deltamethrin, permethrin and DDT. These results are in line with previous reports [14–17] from other parts of Gabon especially in Zilé [18], where high proportions of *An. gambiae* were carrying the *kdr* mutations. However, the fact that metabolic resistance was not assessed constitute a limitation to the present study as the differences in KDTs observed between the three insecticides (DDT, permethrin and deltamethrin) who have similar target sites, suggest the involvement of other resistance mechanisms.

## Conclusion

The current study revealed a high level of resistance of *An. gambiae s.s.* in both Lambaréné and Zilé accompanied with a high frequency of the *Vgsc-L1014F* mutations. The resistance level was higher for permethrin and DDT compared to deltamethrin. Therefore, deltamethrin may be a better option for malaria vector control in the Moyen-Ogooué Province. However, the involvement of other resistance mechanisms should be further investigated for the introduction of control measures adapted to the local settings.

## Data Availability

Raw data are archived and available on request from the corresponding author.

## Abbreviations

WHO: World Health Organization
LLIN: Long-lasting insecticidal net
IRS: Indoor Residual Spraying
Vgsc: voltage-gated sodium channel IPTp-SP:intermittent preventive treatment with sulfadoxine-pyrimethamine
OCEAC: Organisation de Coordination pour la lutte contre les Endémies en Afrique Centrale
kdr: Knockdown resistance gene
PBO: piperonyl butoxide
CERMEL: Centre de Recherches Médicales de Lambaréné
DDT: dichlorodiphenyltrichloroethane
DNA: Deoxyribonucleic acid
PCR: Polymerase chain reaction
*SINE200*: Short INterspersed Element
KDT: Knockdown Time
